# Two independent families with de novo whole *APC* gene deletion and intellectual disability: a case report

**DOI:** 10.1186/s13053-024-00297-1

**Published:** 2025-01-08

**Authors:** Moriya Iwaizumi, Terumi Taniguchi, Risa Kojima, Harumo Osawa, Kyota Tatsuta, Mayu Sakata, Satoshi Osawa, Kiyotaka Kurachi, Ken Sugimoto

**Affiliations:** 1 Department of Laboratory Medicine, Hamamatsu University School of Medicine, Hamamatsu, 431-3192 Japan; 2 Clinical and Molecular Genetics Center, Hamamatsu University School of Medicine, Hamamatsu, 431-3192 Japan; 3 Department of Surgery, Hamamatsu University School of Medicine, Hamamatsu, 431-3192 Japan; 4 Department of Endoscopic and Photodynamic Medicine, Hamamatsu University School of Medicine, Hamamatsu, 431-3192 Japan; 5 First Department of Medicine, Hamamatsu University School of Medicine, Hamamatsu, 431-3192 Japan

**Keywords:** Familial adenomatous polyposis, Intellectual disability, Whole *APC* deletion, De novo mutation, *APC* gene, Chromosome 5q deletion

## Abstract

**Background:**

Familial adenomatous polyposis (FAP) is an autosomal dominant colorectal tumour syndrome characterised by the formation of multiple adenomatous polyps throughout the colon. It is important to understand the extracolonic phenotype that characterizes FAP. Most previous case reports of patients with both FAP and intellectual disability (ID) have described deletions in all or part of chromosome 5q, including the *APC* locus. However, it remains unclear whether the ID phenotype in patients with FAP is due to *APC* disruption or another genetic defect in the deleted 5q region.

**Case presentation:**

Patient of family 1 is a 32-year-old woman presented with > 500 colorectal adenomatous polyps, gastric fundic gland polyposis, several duodenal adenomas, and mild intellectual disability (ID). She had no known family history of the FAP phenotype or ID. By copy number trio analysis, a 15.4 Mb interstitial heterozygous de novo deletion including *APC* region was observed in 5q21.2. q22.3. The patient in family 2 was a 29-year-old man with approximately 50 colorectal adenomatous polyps, fundic gland polyposis in the stomach, non-ampullary adenomas in the duodenum, and mild ID. He had no family history of the FAP phenotype or ID. Using copy number trio analysis, a de novo 9.8 Mb heterozygous deletion was identified on 5q22.1. q23.1 which includes the *APC* region.

**Conclusions:**

Based on previous reports and the present study, we narrowed down the 5p deletion region associated with ID in FAP. Further investigation is required to understand ID due to 5q stromal deletion.

## Background

Familial adenomatous polyposis (FAP; MIM#175,100) is an autosomal dominant colorectal tumour syndrome caused by a heterozygous mutation in the APC regulator of the WNT signalling pathway gene (*APC*; MIM*611731) on chromosome 5q22.2 and is characterised by the formation of numerous adenomatous polyps throughout the colon [1]. Because the lifetime risk of colorectal cancer is almost 100% if the colon is not removed, it is important to understand not only the colonic but also the extracolonic phenotypes that are characteristic of FAP to prevent cancer progression. Among extra-colonic phenotypes, physicians may forget to check whether patients have any non-malignant extraintestinal manifestations of FAP, such as osteomas, dental abnormalities, congenital hypertrophy of the retinal pigment epithelium (CHRPE), and desmoid, because these phenotypes are not always life-threatening.

*APC* are involved in the regulation of various cellular processes including axonogenesis, mitosis, cytoskeletal dynamics, cell polarity and apoptosis [2–6]. In addition, some experiments using mice with a mutated *APC* gene have shown learning and memory impairments, autistic-like behaviours, increased repetitive behaviours, reduced social interest as well as abnormal brain morphology and function [7, 8], which indicates that *APC* disruption leads to intellectual disability (ID). However, most previous case reports of patients with both FAP and ID have deletions of all or part of chromosome 5q, which contains the *APC* locus, thereby suggesting that ID is due to a genetic defect in *APC*; it remains unclear whether this is due to another genetic defect due to loss of chromosome 5q [9–19].

We encountered two independent families, each of which had a sporadic proband of FAP with ID, and detected a heterozygous whole *APC* deletion by genetic testing of *APC* in clinical practice; however, it was not clear how wide the deletion extended the whole *APC* region. Here, we describe a monoallelic interstitial deletion of the long arm of chromosome 5, including the *APC* region, and the inheritance pattern of the deletion in each family.

## Case presentation

### Family 1

**Fig. 1:**
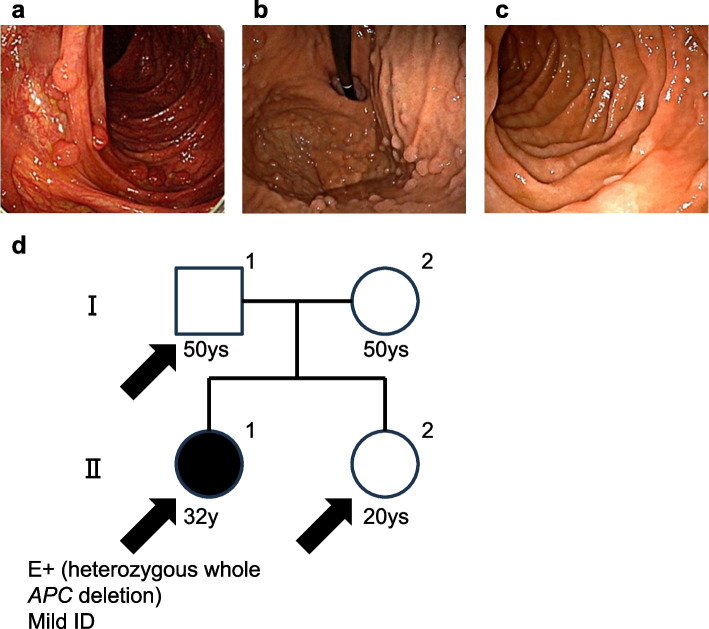
Endoscopic findings and Pedigree chart of Family 1. **a**-**c** Endoscopic findings of the proband (II-1). A complete colonoscopy revealed more than 500 adenomatous polyps in the entire large intestine (**a**) and an additional EGD detected fundic gland polyposis (**b**) in the stomach and several non-ampullary duodenal adenoma (**c**). **d** Pedigree chart of Family 1

A 32-year-old woman consulted a gastroenterologist in her hometown because of a positive faecal occult blood test result for colorectal cancer screening without any change in her bowel habits. A complete colonoscopy revealed more than 500 adenomatous polyps throughout the large intestine, and additional oesophagogastroduodenoscopy (EGD) detected fundic gland polyposis in the stomach and several non-ampullary duodenal adenomas (Fig. 1a-c). As shown in her family pedigree, she had no family history of colonic polyposis or colorectal cancer, despite the typical features of FAP (Fig. 1d). The patient had mild intellectual disability without developmental delay or neurogenic abnormalities. None of her siblings reported any health or behavioural problems or ID. After genetic counselling, she underwent Sanger sequencing and Multiplex Ligation-dependent Probe Amplification (MLPA) for *APC*, and a heterozygous deletion in the entire *APC* region was detected. After the results of genetic testing were disclosed at the second genetic counselling session, the patient underwent total colectomy.

### Family2

**Fig. 2:**
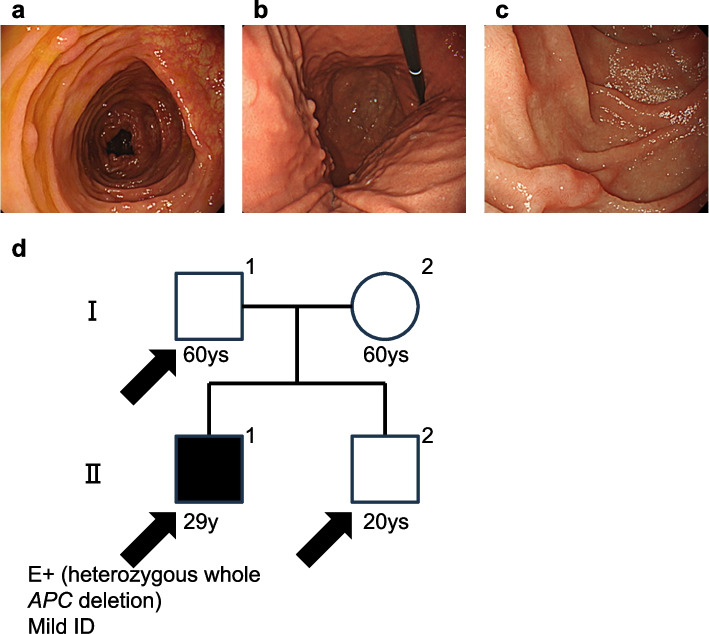
Endoscopic findings and Pedigree chart of Family 2. **a**-**c** Endoscopic findings of the proband (II-1). The patient underwent complete colonoscopy and around 50 adenomatous polyps were found in the entire large intestine (**a**). Additionally, fundic gland polyposis in the stomach (**b**) and several non-ampullary adenomas in the duodenum (**c**) were detected by EGD. **d** Pedigree chart of Family 2

A 29-year-old man visited a gastrointestinal clinic with a chief complaint of bloody stools without any other abdominal symptoms. The patient underwent complete colonoscopy, and approximately 50 adenomatous polyps were found throughout the large intestine (Fig. 2a). Additionally, fundic gland polyposis in the stomach and several non-ampullary adenomas in the duodenum were detected on EGD (Fig. 2b-c). He appeared to have FAP based on his clinical phenotype, but had no family members with FAP phenotypes (Fig. 2d). The patient had childhood epilepsy and presented with a mild intellectual disability without developmental delays or neurogenic abnormalities. None of his siblings reported any health or behavioural problems or ID. After genetic counselling, he underwent Sanger sequencing and MLPA for *APC*, and a heterozygous deletion in the entire *APC* region was detected. After the genetic diagnosis of FAP, surveillance using annual gastrointestinal endoscopy was initiated.

### Copy Number Analysis by CytoScan HD™ array

**Fig. 3:**
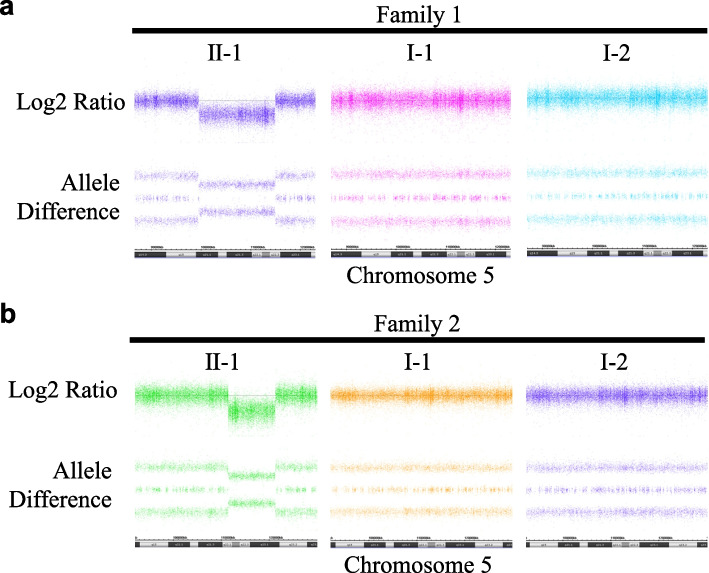
Copy Number Analysis by CytoScan HD™ array. a In the proband of family 1, a 15.4 Mb heterozygous deletion was observed on chromosome 5 (99,418,284–114,816,313), which includes *APC* region (II-1) and the heterodeletions were not found in both parents the probands (I-1 and I-2). **b** In the proband of family 2, a 9.8 Mb heterozygous deletion was identified on chromosome 5 (111,350,204–121,173,067), which includes *APC* region (II-1), and the heterodeletions were not found for both parents (I-1 and I-2)

Sanger sequencing and MLPA revealed that the probands in both families harboured a heterozygous deletion in at least the entire *APC* gene region. However, the extent to which this deletion extends beyond the *APC* region could not be determined using these tests. To answer this question, we used a CytoScan HD™ array (Thermo Fisher Scientific, catalogue no. 901835), which had the highest SNP density. This 1 × 2.6 M platform contains 750,000 unique SNPs and 1.9 million oligonucleotide probes, with an average SNP probe spacing of 200 per megabase (Mb). High-density (HD) assays were performed according to the manufacturer’s protocol. Briefly, 250 ng of patient DNA was digested with Nsp1, amplified with TITANIUM Taq DNA polymerase, fragmented with the Affymetrix fragmentation reagent (Thermo Fisher Scientific), and labelled with biotin end-labelled nucleotides. DNA was hybridised to the microarray for 16 h, washed on an Affymetrix GeneChip Fluidics Station 450 (Thermo Fisher Scientific), stained with Affymetrix GeneChip Stain Reagents, and scanned using a GeneChip Scanner 3000 7G (Thermo Fisher Scientific). Data analysis was performed using Chromosome Analysis Suite software version 4.3 (Thermo Fisher Scientific). In the proband of family 1, a 15.4 Mb heterozygous deletion was observed on 5q21.2. q22.3 (chromosome 5:99,418,284–114,816,313), which includes the *APC* region (Fig. 3a, II-1) and the heterodeletions were not found in both parents of the probands (Fig. 3a, I-1, I-2). A 9.8 Mb heterozygous deletion was identified in the proband of family 2 on 5q22.1. q23.1 (chromosome 5:111,350,204–121,173,067), which includes the *APC* region (Fig. 3b, II-1) without the heterodeletions for both parents (Fig. 3b, I-1, I-2). Based on these results, we concluded that the genomic changes in the probands of each family were de novo heterodeletions.

## Discussion and conclusions

**Fig. 4:**
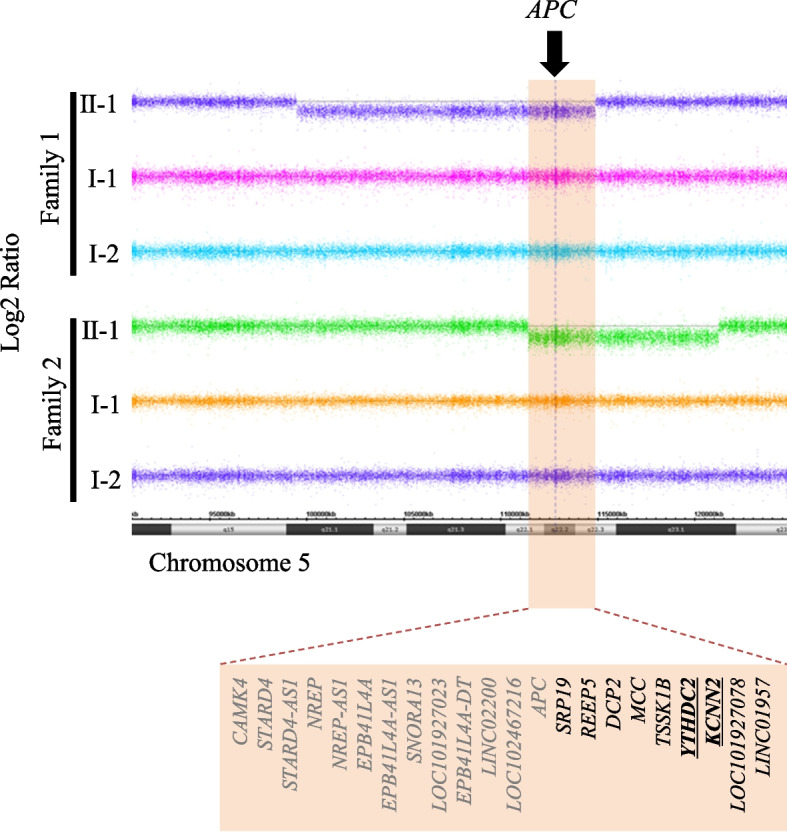
Candidate genes that might cause intellectual disability in the case of heterozygous deletions. 22 OMIM-registered genes (*CAMK4*, *STARD4*, *STARD4-AS1*, *NREP*, *NREP-AS1*, *EPB41L4A*, *EPB41L4A-AS1*, *SNORA13*, *LOC101927023*, *EPB41L4A-DT*, *LINC02200*, *LOC102467216*, *APC*, *SRP19*, *REEP5*, *DCP2*, *MCC*, *TSSK1B*, *YTHDC2*, *KCNN2*, *LOC101927078*, and *LINC01957*) were in the common deletion region (3.47 Mb) observed in our study for the two probands. Yamaguchi et al. reported a patient with FAP with heterodeletions in 13 gene loci (*CAMK4*, *STARD4*, *STARD4-AS1*, *NREP*, *NREP-AS1*, *EPB41L4A*, *EPB41L4A-AS1*, *SNORA13*, *LOC101927023*, *EPB41L4A-DT*, *LINC02200*, *LOC102467216*, *APC*) [21]. Among the remaining nine genes, the genes with a probability of loss-of-function intolerance (pLI) due to gnomAD of 0.9 or higher were *YTHDC2* and *KCNN2*. *KCNN2* is reportedly associated with ID [22], while *YTHDC2* is not

Patients with 5q heterozygous deletions exhibit various phenotypes that are not yet fully linked to a specific genetic cause. The most common clinical features include predisposition to cancer, ID, dysmorphic facies, and neurodevelopmental delay. Privitera et al. reported a case of FAP and ID with a stromal 19.85 Mb deletion in chromosome 5q, including the *APC* region and reviewed previous 12 cases reported or registered in the Decipher database [20]. The overlap of all the deleted regions was approximately cytobands 5q22.1q23.1 (7.77 Mb). In this study, we report two patients with ID, each from an independent family, with a large interstitial deletion at 5q21.2. q22.3 (Family 1), and 5q22.1. q23.1 (Family 2), which includes the *APC* region. In our study, we were able to narrow down the common deletion regions reviewed by Privitera et al.. 22 OMIM-registered genes (*CAMK4*, *STARD4*, *STARD4-AS1*, *NREP*, *NREP-AS1*, *EPB41L4A*, *EPB41L4A-AS1*, *SNORA13*, *LOC101927023*, *EPB41L4A-DT*, *LINC02200*, *LOC102467216*, *APC*, *SRP19*, *REEP5*, *DCP2*, *MCC*, *TSSK1B*, *YTHDC2*, *KCNN2*, *LOC101927078*, and *LINC01957*) were in the common deletion region (3.47 Mb) observed in our study for two probands (Fig. 4). Interestingly, the narrowed common region is completely included the region that Privitera et al. reviewed. Yamaguchi et al. reported a patient with FAP without ID who had a large genomic deletion in chromosome 5q22.1–22.2, which included *CAMK4*, *STARD4*, *STARD4-AS1*, *NREP*, *NREP-AS1*, *EPB41L4A*, *EPB41L4A-AS1*, *SNORA13*, *LOC101927023*, *EPB41L4A-DT*, *LINC02200*, *LOC102467216*, and *APC* [21]. Based on the findings of Yamaguchi et al. and our present report, we hypothesised that the following genes may cause ID when a large heterozygous deletion involving the *APC* region occurs: *SRP19*, *REEP5*, *DCP2*, *MCC*, *TSSK1B*, *YTHDC2*, *KCNN2*, *LOC101927078*, *LINC01957*. Among these, the genes with probability by gnomAD of being loss-of-function intolerant (pLI) of 0.9 or higher were *YTHDC2* and *KCNN2*.

*KCNN2* (MIM*605879) encodes the potassium calcium-activated channel subfamily N member 2, which is involved in membrane excitability. According to the OMIM database, this gene is associated with myoclonic dystonia type 34 (MIM#619724) and neurodevelopmental disorders, with or without various movement and behavioural abnormalities (MIM#619725), both of which have autosomal dominant inheritance. Intolerance to haploinsufficiency of *KCNN2* is strongly supported by significant depletion in truncating variants of this gene in gnomAD [pLI = 0.99; putative loss-of-function observed/expected = 0.09 (0.04–0.27)]. Mochel et al. demonstrated that *KCNN2* variants underlie dominant channelopathy, which is characterised by developmental delay and movement disorders [22]. Interestingly, all 11 patients with *KCNN2* variants had an ID. *YTHDC2* (MIM*616530) encodes an RNA helicase that is involved in RNA processing and metabolism. The intolerance of *YTHDC2* to haploinsufficiency was supported by the significant depletion of truncating variants of this gene in gnomAD [pLI = 0.99; putative loss-of-function observed/expected = 0.06 (0.03–0.13)]. However, there are no reports associated with *YTHDC2* and ID, nor has this been confirmed, even in the OMIM database. Based on current knowledge, this study confirmed that *KCNN2* is most likely an autosomal dominant candidate gene for ID associated with 5q interstitial deletions.

In conclusion, we encountered two independent families with FAP with de novo heterozygous 5q deletions involving the *APC* region that could narrow down the 5p deletion region associated with ID. Although rare, patients with typical phenotypes of both FAP and ID require further investigation to understand ID caused by 5q stromal deletion.

## Data Availability

No datasets were generated or analysed during the current study.

## Abbreviations

FAP: Familial adenomatous polyposis
APC: APC regulator of the WNT signalling pathway gene
ID: Intellectual disability
EGD: Oesophagogastroduodenoscopy
MLPA: Multiplex Ligation-dependent Probe Amplification
